# Non-electrostatic interactions associated with aggregate formation between polyallylamine and *Escherichia coli*

**DOI:** 10.1038/s41598-023-42120-2

**Published:** 2023-09-08

**Authors:** Masatoshi Nakatsuji, Natsuki Sato, Shiho Sakamoto, Koji Watanabe, Yoko Teruuchi, Minoru Takeuchi, Takashi Inui, Hideki Ishihara

**Affiliations:** 1 Research and Development Headquarters, Nitto Boseki Co., Ltd., 2-4-1 Kojimachi, Chiyoda-ku, Tokyo, 102-8489 Japan; 2 Laboratory of Biological Macromolecules, Graduate School of Life and Environmental Sciences, Osaka Prefecture University, 1-1 Gakuen-cho, Naka-ku, Sakai, Osaka 599-8531 Japan; 3 Faculty of Pharmacy, Osaka Medical and Pharmaceutical University, 4-20-1 Nasahara, Takatsuki, Osaka 569-1094 Japan; 4 Specialty Chemicals Division, Nittobo Medical Co., Ltd., 1 Shiojima, Fukuhara, Fukuyama, Koriyama, Fukushima 963-8061 Japan; 5 Present Address: Laboratory of Biological Macromolecules, Graduate School of Agriculture, Osaka Metropolitan University, 1-1 Gakuen-cho, Naka-ku, Sakai, Osaka 599-8531 Japan

**Keywords:** Microbiology techniques, Biomaterials, Biological techniques, Microbiology

## Abstract

Bacterial aggregation by mixing with polymers is applied as pretreatment to identify pathogens in patients with infectious diseases. However, the detailed interaction between polymers and bacteria has yet to be fully understood. Here, we investigate the interaction between polyallylamine and *Escherichia coli* by isothermal titration calorimetry. Aggregation was observed at pH 10 and the binding was driven by favorable enthalpic gain such as the electrostatic interaction. Neither aggregation nor the apparent heat of binding was observed at pH 4.0, despite the strong positive charge of polyallylamine. These results suggest that intermolecular repulsive forces of the abundant positive charge of polyallylamine cause an increased loss of conformational entropy by binding. Non-electrostatic interaction plays a critical role for aggregation.

## Introduction

Synthetic polymers have been applied to various fields because their chemical and physical properties can be modified at pre- or post-polymerization^1,2^. For instance, cationic polymers are widely developed as novel antimicrobial agents that exhibit different growth inhibition mechanisms compared with conventional antibiotics^3,4^. The surface of bacterial cells are negatively charged because of the cell wall and outer membrane components, which include teichoic acids in Gram-positive bacteria, and lipopolysaccharides and phospholipids in Gram-negative bacteria^5^. Therefore, cationic polymers electrostatically bind to them^6^, and disrupt the bacterial cell wall by other properties such as hydrophobic or chelating effects, which inhibits growth^7,8^ or causes cell death^6,9^.

Rapid identification of causative bacteria for the treatment of bloodstream infections, including sepsis, and the administration of optimal antimicrobial agents, lead to lower mortality rates^10–12^. In recent years, technology for the analysis of protein fingerprint signatures generated from whole bacterial cells in matrix-assisted laser desorption ionization time-of-flight mass spectrometry (MALDI-TOF MS) has significantly reduced the time required for bacterial identification in clinical sites^13,14^. For MALDI-TOF MS analysis, it is essential to recover as large a number of bacteria as possible from blood culture bottles from a patient with suspected bloodstream infections^15,16^. We have previously developed a unique pretreatment kit named rapid BACpro® II, which contains one of our proprietary cationic polymers, a polyallylamine hydrochloride (PAA; Supplementary Fig. S1A) copolymer^17^. The PAA copolymer is associated with bacteria, and can efficiently collect them as aggregates under basic condition that are easily visible from positive blood culture bottles^17^. Although it is considered that the formation of these aggregations under basic condition is driven by the electrostatic interactions between cationic polymer and bacteria, and is subsequently caused by the charge neutralization and the bridge of the cationic polymer by the interactions^18,19^, the precise mechanism has not completely understood. Clarification of the aggregation mechanism under basic condition is strongly expected to lead further development of polymers with improved efficacy for collection of bacteria from positive blood culture bottles. The aggregation involves multiple complicated reactions, such as the binding of bacteria and polymers, and their cross-linking. Therefore, quantitative and various analyses are required to elucidate the binding and aggregation mechanisms.

In the present study, to clarify the aggregation mechanism of bacteria by a pretreatment kit including PAA copolymer used under basic condition, we investigated the behavior of PAA for the binding and the aggregate formation to *Escherichia coli* (*E. coli*) under basic condition (pH 10) using isothermal titration calorimetry (ITC), aggregation assay and dynamic light scattering (DLS). The results suggested that both electrostatic interactions between polymers and bacteria and the non-electrostatic interactions associated with the conformational changes in polymers play critical roles for the binding and aggregate formation between PAA and *E. coli*.

## Results

### Thermodynamic characterization of the interaction between PAA and *E. coli*

**Figure 1:**
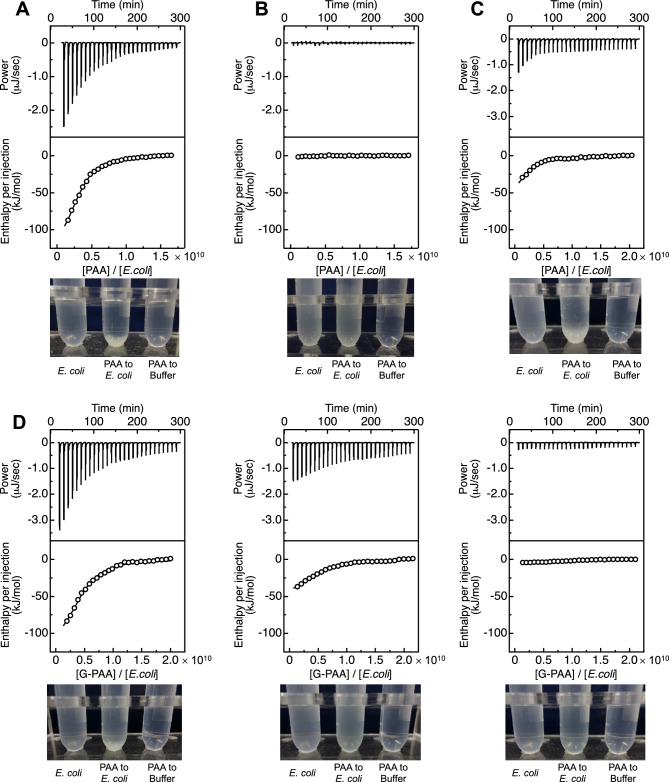
ITC measurements of PAA binding with *E. coli* at (**A**) pH 10 and (**B**) pH 4.0. Thermograms for the titration of PAA into *E. coli* are shown in the upper panels. Normalized changes in heat after subtraction of the heat of dilution are plotted against the molar ratio ([PAA]/[*E. coli*]) in the lower panels. The one-set of independent binding sites model was used to fit the binding isotherms, and the fitting curves are shown as continuous lines. The lower images show the solutions in ITC cells after the measurements. (**C**) ITC measurements of PAA binding with *E. coli* in 150 mM NaCl at pH 10. (**D**) ITC measurements of 19.7% G-PAA (left), 37.7% G-PAA (middle), and 59.0% (right) G-PAA binding with *E. coli* at pH 10.

ITC measurements were performed under high and low pH conditions to obtain the thermodynamic parameters on the binding between PAA and *E. coli*. Exothermic reactions were detected and aggregates were observed by titrating PAA into *E. coli* at pH 10 (Fig. 1A). The thermodynamic parameters for the binding of PAA to *E. coli* were obtained by fitting of the thermogram using the one-set of independent binding sites model (Fig. 1A and Table 1). The results revealed that PAA binds to *E. coli* with a dissociation constant (*K*_d_) of 1.26 ± 0.10 µM. In addition, the enthalpy change (Δ*H*) and entropy term (− *T*Δ*S*) for the binding of PAA to *E. coli* were − 131 ± 5.6 and 97.6 ± 5.6 kJ/mol, respectively, which indicates that the enthalpy favorably contributes to the binding. It has been reported that *E. coli* shows a negative zeta potential at pH 2.0–12^20^, and we have also confirmed that *E. coli* used in this study has a constant negative zeta potential of − 35 mV at pH 4.0 and pH 10 (Supplementary Fig. S2). In addition, PAA has a primary amine group that is protonated depending on the pH. Since we could not obtain enough light scattering intensity due to the small size of PAA and calculate the zeta potential of PAA, we measured the zeta potential of PAA with a high degree of polymerization (PAA-high: about two-fold) compared with PAA used in this study. PAA-high showed a positive zeta potential of 3.1 ± 2.2 mV at pH 10, which suggests that PAA used in this study has also a weak positive charge at pH 10 and the electrostatic interactions contribute to the binding between PAA and *E. coli*. On the other hand, according to previous reports^21,22^, the p*K*_a_ of PAA is approximately 9.70; therefore, PAA shows a weak positive charge with partial protonation of the amine group at pH 10. To investigate the effect of positive charge of PAA on the binding to *E. coli*, ITC measurements were performed at pH 4.0. Interestingly, the apparent heat of binding was observed to be negligible, showing only background signal by the heat of dilution when PAA was titrated into a buffer (Fig. 1B). Aggregates were not observed in the reactant cell (Fig. 1B). These results suggested that PAA did not bind to *E. coli* at pH 4.0. ITC measurements were also performed in the presence of 150 mM NaCl at pH 10 to inhibit electrostatic interactions. As a result, the apparent heat by titrating PAA into *E. coli* was reduced by the addition of NaCl (Fig. 1C and Table 1).

**Table 1:** Thermodynamic parameters for the binding between polymer and *E. coli* at pH 10 obtained by ITC.

	*n*	*K*_d_ (µM)	Δ*G* (kJ/mol)	Δ*H* (kJ/mol)	− *T*Δ*S* (kJ/mol)
PAA	3.2 × 10^9^	1.26 ± 0.10	− 33.7 ± 0.2	− 131.3 ± 5.6	97.6 ± 5.6
PAA + NaCl	2.0 × 10^9^	2.79 ± 0.45	− 31.7 ± 0.4	− 72.7 ± 14.1	40.9 ± 14.1
19.7% G-PAA	3.9 × 10^9^	2.22 ± 0.27	− 32.3 ± 0.3	− 123.2 ± 7.7	90.9 ± 7.7
37.7% G-PAA	4.7 × 10^9^	2.55 ± 0.30	− 31.9 ± 0.3	− 53.6 ± 3.3	21.7 ± 3.3
59.0% G-PAA	N.D	N.D	N.D	N.D	N.D

To confirm the electrostatic interaction, we further performed the experiments with G-PAA; Glycolylation of the amino group of PAA. Three types of G-PAA with different degrees of substitution (19.7%, 37.7% and 59.0%) were synthesized and confirmed by FT-IR, NMR, and acid–base titration measurement (Supplementary Figs. S3 and S4). In addition, the diameter of G-PAA was similar to that of PAA (Supplementary Table S1). At pH 10, the apparent heat by titrating G-PAA into *E. coli* was reduced with respect to the degree of substitution of the primary amine group, and was not observed in 59.0% G-PAA (Fig. 1D). The Δ*H* values obtained by titrating G-PAA into *E. coli* were negative, and increased from − 131 to − 54 kJ/mol with increased substitution. On the other hand, − *T*Δ*S* showed positive values and decreased from 97.6 to 21.7 kJ/mol with an increased amount of substitution. The binding stoichiometry (*n*) and *K*_d_ values were comparable, regardless of the amount of substitution (Table 1). These results showed that the electrostatic interaction of the primary amine group of PAA is associated with the binding between PAA and *E. coli*.

### Effect of PAA and *E. coli* interaction on aggregation

**Figure 2:**
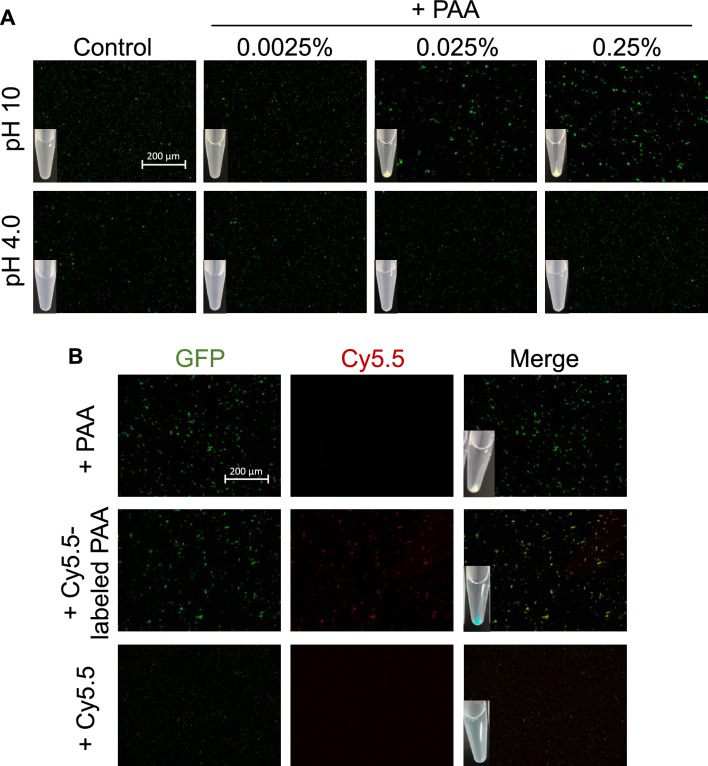
Fluorescence microscopic images of GFP-expressed *E. coli* incubated with PAA. (**A**) GFP-expressed *E. coli* was incubated with PAA (0, 0.0025, 0.025, and 0.25% w/v) at pH 10 or 4.0 for 3 min. Scale bar: 200 μm. The aggregates were visually observed after weak centrifugation at 2000×*g* for 10 s. (**B**) GFP-expressed *E. coli* was incubated with 0.25% PAA or Cy5.5-labeled PAA at pH 10 for 3 min. The fluorescent signals of GFP show the co-localization with Cy5.5-labeled PAA. Left: GFP. Middle: Cy5.5. Right: Merged image. Scale bar: 200 μm.

The effect of the interaction between PAA and *E. coli* on the formation of aggregates was investigated using fluorescence microscopy (Fig. 2A). The GFP-expressed *E. coli* was used in this assay. Aggregates of GFP-expressed *E. coli* formed by mixing with PAA were observed at pH 10, but not at pH 4.0. In addition, the formation of aggregates at pH 10 was increased in a PAA concentration-dependent manner. Next, the primary amine of PAA was labeled with Cy5.5 and incubated with GFP-expressed *E. coli* at pH 10 verify whether PAA was co-localized with *E. coli* in the aggregates or not (Fig. 2B). The Cy5.5 labeling of PAA was partially performed to leave the primary amine for interaction with *E. coli* (3% of amino group of PAA were labeled with Cy5.5.). The red signal derived from Cy5.5-labeled PAA co-localized with the GFP fluorescence signal from *E. coli* indicated that both PAA and *E. coli* were located in the aggregates. These results showed that the binding of PAA to *E. coli* was directly associated with the formation of aggregates.

### Size distribution analysis of PAA

**Figure 3:**
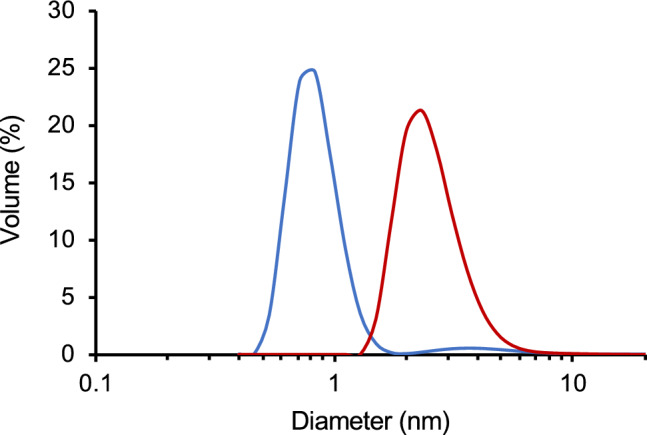
Size distribution of PAA at pH 10 (blue) and 4.0 (red) from DLS measurements.

Finally, to clarify the difference in the binding behavior of PAA to *E. coli* at different pH, the size distributions of PAA were measured at pH 10 and 4.0 (Fig. 3). At pH 10, PAA showed a bimodal size distribution with one peak in the range 0.6–1.5 nm and the other peak in the range 2.0–6.0 nm. The majority of PAA had a hydrodynamic diameter of 0.88 nm (Table 2). On the other hand, a single peak of PAA with an average hydrodynamic diameter of 2.53 nm was obtained at pH 4.0, which was approximately three times larger than that at pH 10 (Table 2). The p*K*_a_ value of PAA is 9.70; therefore, PAA has a high positive charge with protonation of the amine group at pH 4.0, compared with that at pH 10. Therefore, the pH-dependent conformational change of PAA is considered to be caused by intermolecular repulsive forces of the positive charge. Considering that PAA did not bind to *E. coli* at pH 4.0, the conformational change of PAA may thus be associated with the binding.

**Table 2:** Average hydrodynamic diameter of PAA at pH 10 and 4.0.

pH	Diameter (nm)
10	0.88 ± 0.22
4.0	2.53 ± 0.25

## Discussion

We investigated the binding and aggregation profile between PAA and *E. coli*, with focus on the thermodynamic properties and conformation of the polymer. The results suggest that both electrostatic and non-electrostatic interactions, such as the conformational change of PAA, are associated with the binding and aggregation between PAA and *E. coli*.

Most of the research on cationic polymers has focused on functions such as the bacterial aggregate-forming ability and the antimicrobial activity; however, there have been no detailed studies on the mechanism^23,24^. Louzao et al. investigated the bacterial aggregate-forming ability of cationic co-polymers that consisted of a tertiary amine group and catechol moiety using microscopy, and reported the importance of the balance between charge and the hydrophobicity of the side-chain for their functionality^5^. However, their approaches only indirectly investigated the parameters involved in the formation of aggregates with bacteria, and it has remained unclear how each parameter affects aggregation. On the other hand, Yuan et al. reported that electrostatic interactions were associated with the binding between the cationic polymer and *E. coli* by ITC measurements; however, the aggregation mechanism with respect to the thermodynamic parameters was not discussed^23^. Here, we successfully achieved direct measurement of the interaction between cationic polymers and *E. coli* using ITC, and revealed the detailed thermodynamic parameters for their interaction, and the relationship between the interaction and formation of the aggregates. Therefore, the results contribute to elucidation of the detailed binding and aggregation mechanism, and show the utility of ITC measurement for analysis of the interaction between macromolecules and living organisms, such as bacteria and cell lines.

**Figure 4:**
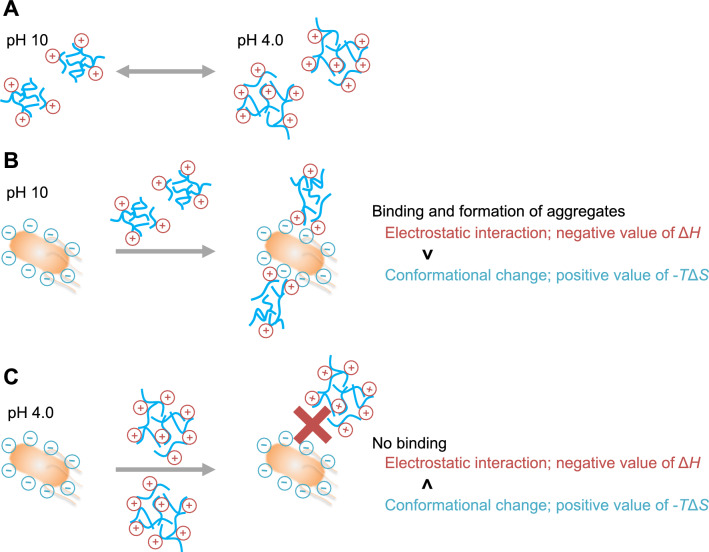
Proposed binding model of PAA to *E. coli*. (**A**) The pH-dependent conformational change of PAA. At pH 10, PAA shows compact structure as compared with that at pH 4.0. (**B**) At pH 10, the positive charge of the amine group in PAA binds to the negatively charged cell wall in *E. coli* through electrostatic interactions, and the structure of PAA becomes more compact. (**C**) At pH 4.0, PAA did not bind to *E. coli*, because the intermolecular repulsive forces of the abundant positive charge cause an increased loss of entropy.

From these results, we propose a model on the binding between PAA and *E. coli* (Fig. 4). PAA exhibits a pH-induced reversible conformational change with protonation of the amine group (Figs. 3 and 4A). On the other hand, *E. coli* shows the similar surface charge behavior at pH 4.0 and pH 10 (Supplementary Fig. S2). At pH 10, PAA has a partial positive charge and compact structure compared with that at pH 4.0 (Fig. 4A), and thus binds to the negatively charged cell wall in *E. coli* through electrostatic interactions, which favorably contributes to binding (negative value of Δ*H*, Table 1 and Fig. 4B). At the same time, the interactions induce further conformational changes of PAA, which adversely affects binding (positive value of − *T*Δ*S*, Table 1 and Fig. 4B). ITC measurements using G-PAA at pH 10 showed a decrease of the enthalpic gains and an increase of Δ*S* that was dependent on the degree of substitution (Table 1). These results suggested that, in the binding between PAA and *E. coli*, the enthalpic gains obtained by the electrostatic interactions are compensated by a loss of entropy. On the other hand, PAA did not bind to *E. coli* at pH 4.0, despite the strong positive charge of PAA and the negative charge of the surface of *E. coli*^20^ (Figs. 1B and 4C). The intermolecular repulsive forces of the abundant positive charge may cause an increased loss of entropy that is larger than the enthalpic gain obtained by the electrostatic interactions (Fig. 4C). Therefore, the results suggest that the binding of PAA to *E. coli* is regulated by the balance of enthalpy-entropy compensation, similar to the typical protein–ligand interaction^25,26^. However, we have not investigated the effect of some other molecules and ions in the solutions such as glycine, acetate, and chloride ion on the interaction between PAA and *E. coli* in this study. Furthermore, the binding and aggregation mechanisms in physiological condition remain unclear. Thus, we should perform the interaction analysis and the aggregation assay under different buffer conditions including physiological pH to clarify their interactions in more detail.

The specificity and regulation of binding and aggregation for various bacterial species using polymers are important for their biomedical use. In particular, it is necessary to distinguish drug-sensitive and drug-resistant pathogens in the clinical diagnosis of infectious diseases. Although antimicrobial susceptibility testing is performed as a traditional method to detect drug-resistant pathogens and guide appropriate antimicrobial agents, it typically takes at least 48 h due to the subculture process from positive blood culture bottles^27^. Therefore, quicker collection from the culture medium to perform antimicrobial susceptibility testing is required. The present results indicate that PAA can bind to *E. coli* and form aggregates at pH 10, but not at pH 4.0 (Figs. 1A,B, and 2A), because of the limitation of conformational change, suggesting that their binding is essential for the aggregate formation. Such pH-dependence in the binding of PAA to *E. coli* may allow for control of the aggregation, and suggests that *E. coli* could be released from the aggregates under acidic conditions. Therefore, although more detailed analysis is required, bacteria that are collected as aggregates from positive blood culture bottles using a polymer may be released by changes in pH or the addition of salt, and could then be quickly applied to antimicrobial susceptibility testing.

In conclusion, we have revealed through direct measurement of the interactions, that non-electrostatic interactions such as the conformational change of polymers play critical roles for the binding of PAA to *E. coli*. Further studies, such as interaction analysis for a Gram-positive bacteria and structural analysis, are required to completely elucidate the binding and aggregation mechanism, although rapid BACpro® II containing PAA derivatives is usable for both Gram-negative and -positive bacteria^17,28–31^. The present results may serve as an alternative development approach with biomedical cationic polymers.

## Methods

### Materials

PAA (M.W. = 1600) was purchased from Nittobo Medical Co., Ltd. (Fukushima, Japan). Methyl glycolate and sodium hydroxide were purchased from Tokyo Chemical Industry Co., Ltd. (Tokyo, Japan). Ampicillin, arabinose, acetate, sodium acetate, glycine, and dimethylformamide were purchased from FUJIFILM Wako Pure Chemical Co., Ltd. (Osaka, Japan). HEPES was purchased from Dojin Chemical Research Institute (Kumamoto, Japan).

### Bacteria strains and growth conditions

The strain *E. coli* BL21 (DE3) was transformed by pGEX 4T-2 vector (Cytiva, Ma, USA) and grown in LB broth containing 100 μg/mL of ampicillin at 37 °C. The GFP-expressed *E. coli* (pGLO™ Bacterial Transformation Kit; Bio-Rad Laboratories, CA, USA) was used for the bacterial aggregation assay, and grown in LB broth containing 1% arabinose and 100 μg/mL of ampicillin at 37 °C. At the end of the exponential growth phase, these bacteria were centrifuged and washed three-times with 100 mM sodium acetate (pH 4.0) or 100 mM glycine sodium hydroxide (pH 10). The bacterial suspension was adjusted by measuring the optical density at a wavelength of 600 nm (OD_600_).

### Isothermal titration calorimetry

ITC experiments were performed with a calorimeter (MicroCal VP-ITC, Malvern Instruments Ltd., UK) at 25 °C. PAA (0.02%, w/w, 125 μM) or glycolylated PAA (19.7% G-PAA: 0.032%, w/w, 156 μM; 37.7% G-PAA: 0.037%, w/w, 157 μM; 59.0% G-PAA: 0.043%, w/w, 157 μM) in a syringe was titrated into *E. coli* solution (OD_600_ = 1.0) in the cell. The volume was 10 μL for each injection, and the cell was continuously stirred at 90 rpm. The corresponding heat of dilution of PAA titrated into the buffer was used to correct the data. The thermodynamic parameters were evaluated using the one-set of independent binding sites model supplied by MicroCal Origin 7.0 software, which each binding site is assumed to show the identical affinity and enthalpy change^25^. The molarity of bacteria was assumed to be calculated using the equation (number of *E. coli* in solution)/(6.02 × 10^23^)^32^.

### Synthesis of G-PAA

The amino group of PAA was substituted with methyl glycolate (Supplementary Figs. S1B and S5)^33^ as follows. Methyl glycolate was reacted to PAA solution with stirring overnight and the resultant mixture was added sodium hydroxide solution, evaporated on a rotary evaporator to remove methanol, and dialyzed to remove by-product sodium glycolate. The degree of substitution of the primary amine group was changed by the reaction condition of methyl glycolate, PAA solution, and sodium hydroxide solution (detail condition was described in the legend of Supplementary Fig.S5.).

### Labeling of PAA

The Cy5.5 labeling of PAA was performed using Cy5.5 NHS ester (Lumiprobe Corporation, MD, USA). PAA (0.1%, w/w, 625 μM) in 100 mM HEPES buffer (pH 7.0, 270 μL) was mixed with Cy5.5 NHS in dimethylformamide (6.25 mM, 30 μL). The mixture was stirred at room temperature for 3 h then 800 μL of 100 mM glycine sodium hydroxide (pH 10) was added to the mixture to stop the reaction for the aggregation assay.

### Bacterial aggregation assay

The suspension of GFP-expressed *E. coli* (OD_600_ = 1.0, 1400 μL) was incubated with PAA (0.0025%, 0.025%, and 0.25%, w/w, 300 μL) or Cy5.5-labeled PAA (0.025%, w/w, 300 μL) in each buffer at room temperature for 3 min. The mixture was observed using fluorescence microscopy (BZ-X710, Keyence, Osaka, Japan).

### Dynamic light scattering

The size distribution of the polymer was measured by DLS (Zetasizer Nano ZS, Malvern Instruments Ltd., UK) at 25 °C. The polymer was diluted in 100 mM sodium acetate (pH 4.0) or 100 mM glycine sodium hydroxide (pH 10).

### Supplementary Information

Supplementary Information.

## Data Availability

All data generated during this study are included in this published article and its Supplementary Information files.
