# Supplementary material for: Non-electrostatic interactions associated with aggregate formation between polyallylamine and Escherichia coli
Source: Sci Rep. 2023 Sep 8;13:14793. doi: 10.1038/s41598-023-42120-2 (PMC10491771; doi:10.1038/s41598-023-42120-2)
Supplement: Supplementary file 1 — Supplementary Information. [file 41598_2023_42120_MOESM1_ESM.pdf]

Non-electrostatic interactions associated with aggregate formation between polyallylamine and *Escherichia coli*

Masatoshi Nakatsuji[1, 2, †], Natsuki Sato[1], Shiho Sakamoto[2], Koji Watanabe[3], Yoko Teruuchi[3], Minoru Takeuchi[1], Takashi Inui[2], Hideki Ishihara[1]

Supporting Information

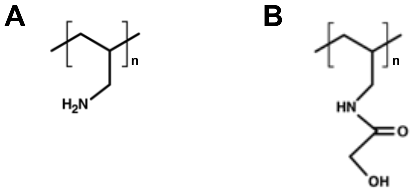

Supplementary Figure S1. Chemical structures of (A) PAA and (B) G-PAA.

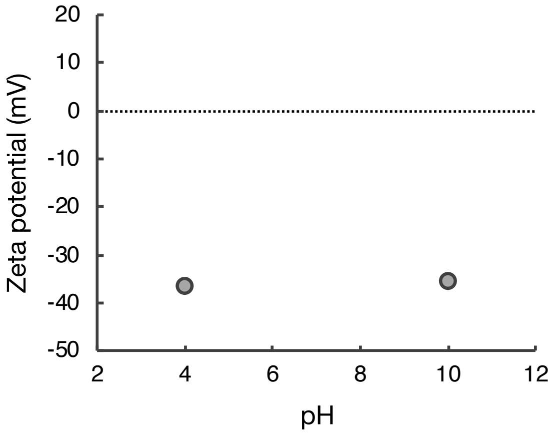

Supplementary Figure S2. Zeta potential of *E. coli* at pH 10 and 4.0.

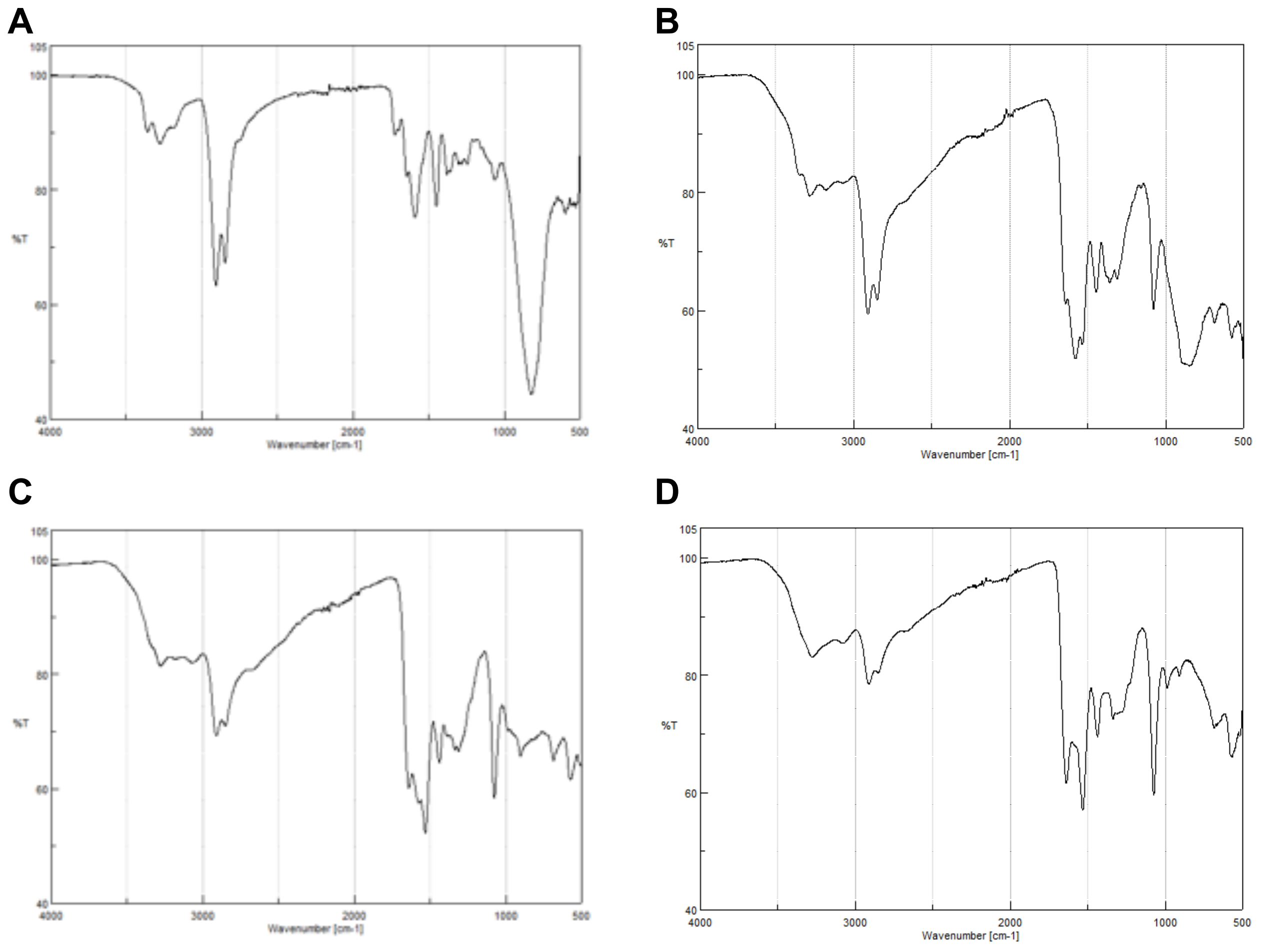

Supplementary Figure S3. FT-IR spectra for (A) PAA, and (B) 19.7% G-PAA, (C) 37.7% G-PAA, and (D) 59.0% G-PAA. The absorption peak at 1640 cm^{-1} was attributed to the amido groups (C=O) and the peak at 1070 cm^{-1} was attributed to carbon-hydroxyl bonding (C-OH). These absorption peaks increased with the degree of substitution.

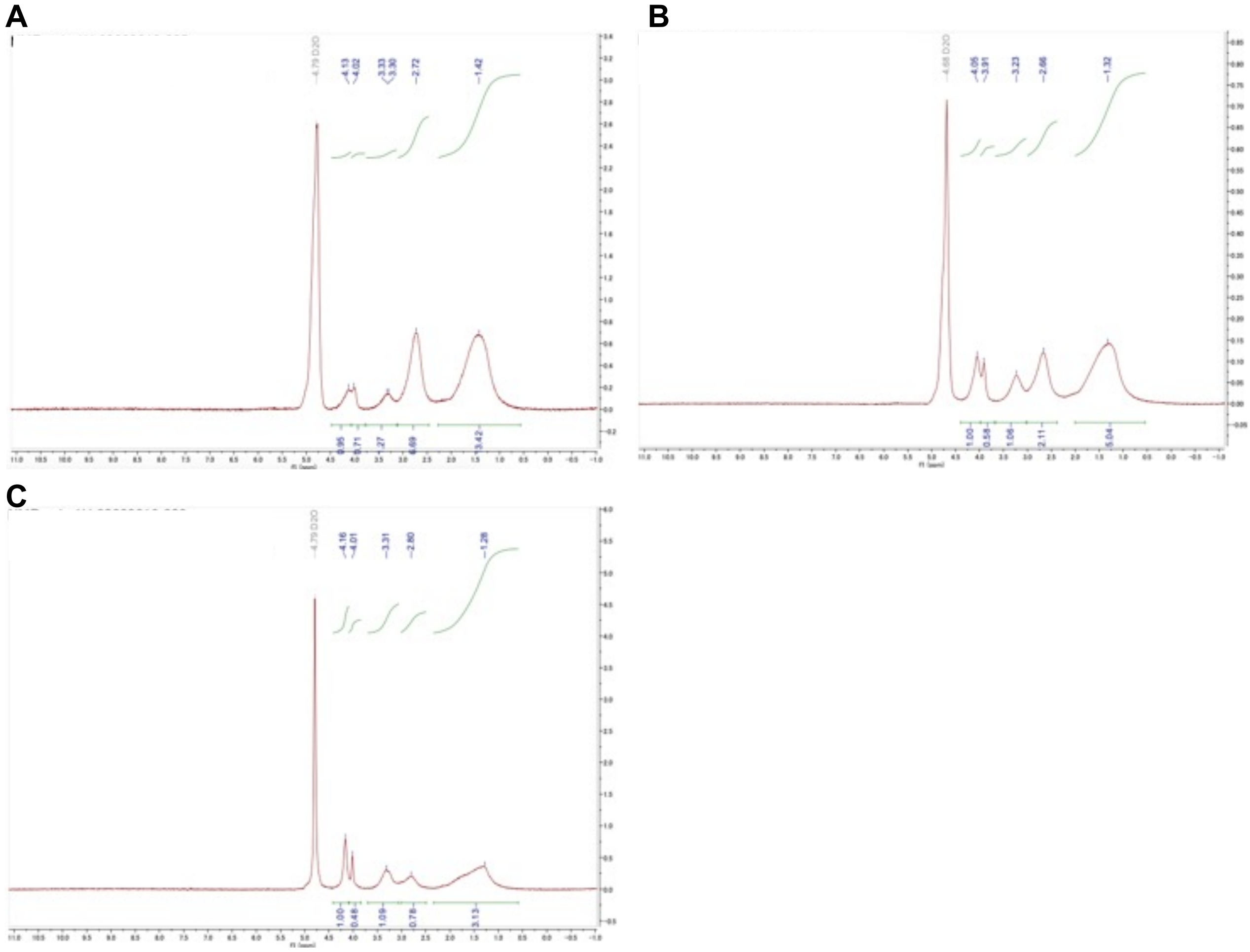

Supplementary Figure S4. ^{1}H-NMR spectra for (A) 19.7% G-PAA, (B) 37.7% G-PAA, and (C) 59.0% G-PAA. The singlet of 2.75 ppm represents methylene group in the side chain of PAA, while the singlet of 3.25 ppm represents methylene group adjacent to the terminal hydroxyl group of G-PAA. The degree of substitution calculated by the ratio between the peak areas were 16.0%, 33.4%, and 58.3%, which are in good agreement with that calculated by acid-base titration measurements.

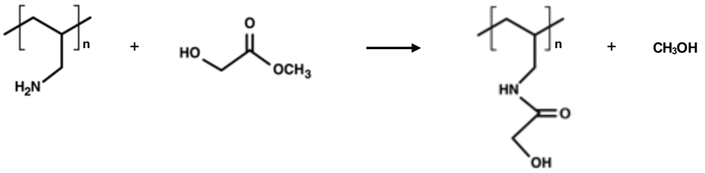

Supplementary Figure S5. Scheme of the synthesis of G-PAA. The degree of substitution of the primary amine group was changed by the reaction condition of methyl glycolate (25% substituted G-PAA:96%, 7.51 g, 0.08 mol; 50% substituted G-PAA: 96%, 16.51 g, 0.18 mol; 75% substituted G-PAA: 96%, 30.03 g, 0.32 mol), PAA solution (25% substituted G-PAA: 15%, 121.41 g, 0.32 mol; 50% substituted G-PAA: 15%, 121.41 g, 0.32 mol; 75% substituted G-PAA: 15%, 121.41 g, 0.32 mol), and sodium hydroxide solution (25% substituted G-PAA: 2.72 g, 0.017 mol; 50% substituted G-PAA: 25%, 10.60 g, 0.07 mol; 75% substituted G-PAA: 25%, 16.41 g, 0.10 mol). The synthesized G-PAA samples were analyzed using Fourier transform infrared spectroscopy (FT-IR; FT/IR-4700, Jasco, Tokyo, Japan), and NMR spectroscopy (NMReady 60 Pro nanalysis-X252, Tokyo instruments, Inc., Tokyo, Japan). The amino group concentration of G-PAA was determined by acid-base titration measurement using an autotitrator (GT-100, Mitsubishi Chemical, Tokyo, Japan). The degree of G-PAA substitution was calculated with the following equation:

$$\text{Substitution (\%)} = (W1 - W2)/115.13/\{W2/57.096 + (W1 - W2)/115.13\}\times 100, \qquad (1)$$

where W1, W2, 115.13, and 57.096 are the solid concentration of G-PAA, the concentration of amino group of G-PAA, the molecular weight of G-PAA, and the molecular weight of PAA, respectively.

Table S1. Average hydrodynamic diameter of G-PAA at pH 10.

The degree of substitution	Diameter (nm)
0%	0.88 ± 0.22
19.7%	1.03 ± 0.32
37.7%	0.89 ± 0.18
59.0%	0.83 ± 0.22
